# Miniaturized catalysis: monolithic, highly porous, large surface area capillary flow reactors constructed *in situ* from polyhedral oligomeric silsesquioxanes (POSS)†
†Electronic supplementary information (ESI) available. See DOI: 10.1039/c5cy00510h



**DOI:** 10.1039/c5cy00510h

**Published:** 2015-06-17

**Authors:** P. Scholder, I. Nischang

**Affiliations:** a Institute of Polymer Chemistry , Johannes Kepler University Linz , A-4060 , Leonding , Austria . Email: ivo.nischang@jku.at

## Abstract

A single-step molding process utilizing free-radical cross-linking reaction of vinyl POSS in microliter-sized dimensions leads to hierarchically-structured, mechanically robust, porous hybrid structures. Functional variants show excellent performance in Suzuki-type coupling reactions. Due to their small volume, long-term operational robustness, and potential chemical diversity, these materials are promising candidates for catalyst screening applications.

## Abstract 2

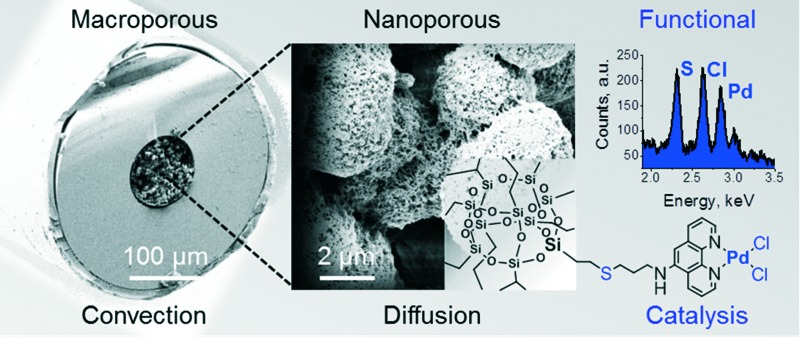
A single-step molding process utilizing free-radical cross-linking reaction of vinyl POSS in microliter-sized dimensions leads to hierarchically-structured, mechanically robust, porous hybrid structures tailored for catalytic applications.

Palladium-based catalytic systems for cross-coupling have been pioneered by Richard F. Heck, Ei-ichi Negishi and Akira Suzuki.^1^ Due to their recognized importance and the inception of a multitude of possibilities emerging therefrom, they have stimulated a great deal of research in the academic and industrial communities.^2^ A recent outreach for related carbon–carbon coupling reactions can be found in the development of advanced materials.^3^

While homogeneous catalytic systems provide high product yields,^4^ their inherent disadvantage is in the necessity to separate products from the catalyst.^5^ This situation has spurred developments in heterogeneous catalytic systems with examples reporting packed bed reactors,^6^ monolithic flow-through formats with silica-based materials^7^ and organic (monolithic) polymers.^8^ Notwithstanding, we note that the catalytic reaction is often quasi-homogeneous in nature.^9^

The recent developments in micro-engineering demand the utilization of miniaturized formats allowing the reduction in solvent consumption, fast heat and mass transfer, as well as continuous operation with the option of massive parallelization.^10^ Such systems allow the use of minute catalyst amounts for screening experiments before up-scaling and producing fine chemicals on a larger scale.

Polyhedral oligomeric silsesquioxanes (POSS) with the formula (RSiO_1.5_)_*n*_ are nano-building blocks have already been used for the creation of advanced porous materials.^11^ We have demonstrated that vinyl POSS cages can be woven into (bulk) porous monolithic networks directly *via* a free-radical initiated *in situ* reaction in suitable porogenic diluents leading to large specific surface area sorbents.^11*c*^ Such hybrid porous materials have not yet entered the micro-engineering arena but possess the option for scaling due to the *in situ* preparation. An important asset of these porous materials is the tailorable amount of tightly tethered vinyl functionality on their internal structure.^11*c*^

We herein communicate our first results in a single-step creation of such large surface area hybrid materials in 100 μm I.D. fused-silica capillaries covalently anchored to the confining capillary wall. This is followed by straightforward functionalization of their internal porous structure to chelate palladium utilized for Suzuki-type coupling reactions.

The internal surface of the 100 μm I.D. fused-silica capillaries was first decorated with pendant methacrylate functionality (for detail see the ESI†).^12^ The porous hybrid monolith precursor mixture was prepared by weighing 20% vinyl POSS as monomer, 51% tetrahydrofuran, and 29% poly(ethylene glycol) 200 as porogenic solvents (all w/w). The precursor mixture additionally contained azobisisobutyronitrile (16 wt% with respect to the vinyl POSS). The homogeneous solution was filled in the capillary mold by means of a syringe. Subsequently, both ends of the capillary were sealed with rubber stoppers and immersed in a water bath with temperature at 60 °C. After monolith formation, the capillary was flushed with tetrahydrofuran to remove the porogenic solvents and initiator.

Fig. 1 shows the sample SEM images of the hybrid pristine monolith. Its pore space possesses a hierarchical structure and the structure is covalently attached to the fused-silica capillary inner wall. A dry-state Brunauer–Emmett–Teller (BET) surface area of 898 m^2^ g^–1^ together with an appreciable dry-state Barrett–Joyner–Halenda (BJH) mesopore volume of 0.4 cm^3^ g^–1^ (Fig. S1†) of the prepared bulk material clearly support the existence of a large surface area, hierarchical porous structure.

**Fig. 1:**
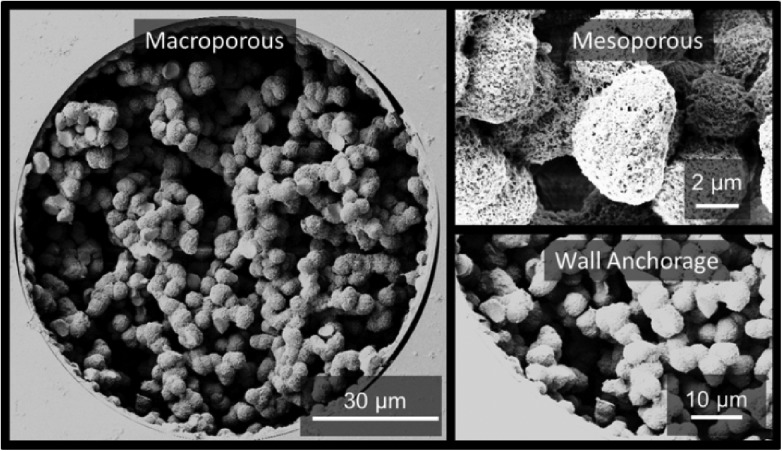
The scanning electron microscopy (SEM) images of the large surface area vinyl POSS hybrid pristine polymer. Cross-section of monolithic hybrid polymer incorporated in 100 μm I.D. fused silica capillaries clearly showing the macroporous structure and indication of an appreciable amount of nanopores (<50 nm) further supported by nitrogen adsorption/desorption measurements of the bulk material (Fig. S1†). Seen as well is the anchorage to the fused-silica capillary wall.

In flow operation, the materials showed a linear increase in back pressure at increased flow rates (Fig. S2†), indicating their maintained mechanical integrity. A total porosity of 83% was determined with flow experiments of a small hydrophilic tracer (for detail see the ESI†). The free-radical initiated linking of vinyl POSS as well results in a multiplicity of pendant vinyl groups (Scheme 1).^11*b*^ Two *in situ* modification strategies were explored for immobilizing the desirable phenanthroline ligand to the internal surface of the scaffold, both employing thiol-ene addition as the primary step (Scheme 1).

**Scheme 1:**
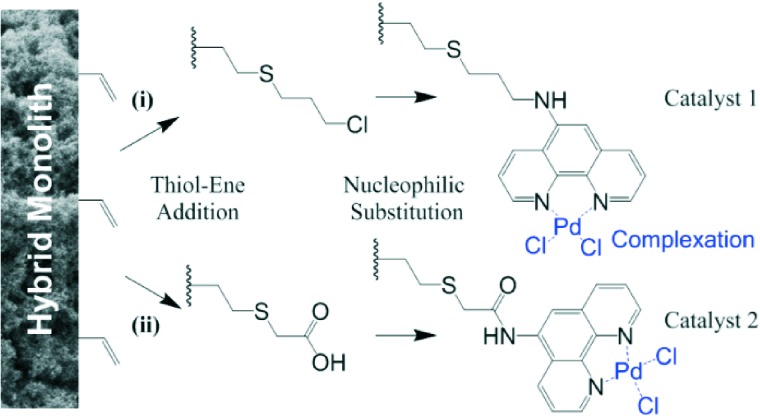
*In situ* functionalization of hybrid porous polymer *via* thiol-ene addition as the primary step with either (i) 3-chloro-1-propane-thiol, or (ii) thioglycolic acid. The following nucleophilic substitution leads to linkage of the 5-amino-1,10-phenanthroline as the chelating functionality for palladium. Further experimental details are provided in the ESI.†

For catalyst **1**, the hybrid monolith was functionalized with 3-chloro-1-propanethiol *via* thiol-ene addition. Then, 5-amino-1,10-phenanthroline was bound *via* nucleophilic substitution under secondary amine formation (Scheme 1, route (i)). For catalyst **2**, the hybrid monolith was modified with thioglycolic acid *via* thiol-ene addition. The acid was then activated to an acyl chloride and 5-amino-1,10-phenanthroline was immobilized *via* amide bond formation (Scheme 1, route (ii)). Both phenanthroline pendant scaffolds were subsequently provided with chelated palladium species by flushing solutions of Pd(MeCN)_2_Cl_2_ complex in acetonitrile through the monoliths. Further details are provided in the ESI.†

To indicate the existence of chelated palladium on the internal structure of the highly porous material according to Scheme 1, the as-prepared and washed reactors were analyzed by energy-dispersive X-ray spectroscopy (EDX) on their cross-sections. The results in Fig. 2 clearly confirm the existence of sulfur, indicating successful thiol-ene addition, as well as Pd and Cl functionalities indicating successful Pd chelation for catalysts **1** and **2** (Scheme 1). These signals are totally absent for the pristine material.

**Fig. 2:**
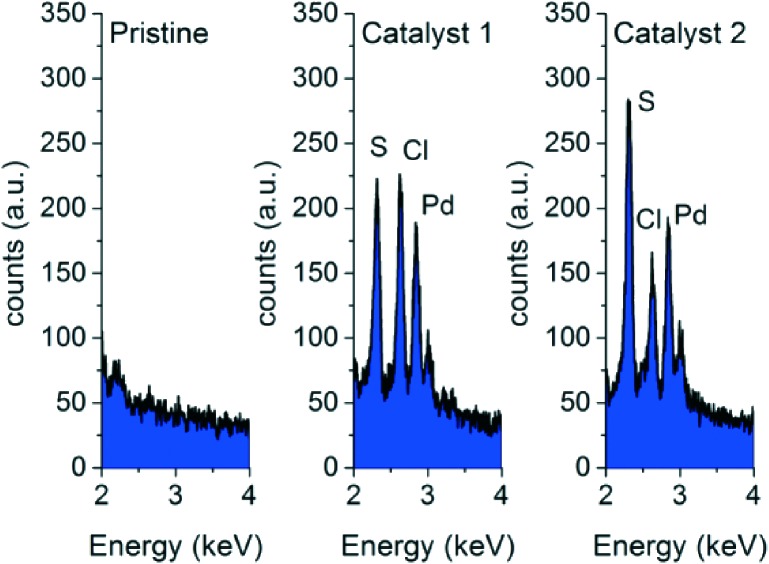
Energy-dispersive X-ray spectroscopy (EDX) analysis of cross-sections of porous materials with the pristine and reactor variants (Scheme 1).

The reaction of iodobenzene with *p*-tolylboronic acid in 75/25 acetonitrile/water (%, v/v) was used as a model to judge the reactor performance under typical conditions, *i.e.* at a temperature of *T* = 80 °C and continuous flow. Fig. 3a compares the respective yields of the flow reactor systems originating from the same pristine material. The materials with chelated palladium functionality (catalysts **1** and **2**, Scheme 1) approach greater than 85% quantitative yield after a mere 30 min fluid contact time (Fig. 3a). The shape of the yield–fluid contact time curves of catalysts **1** and **2** is similar. The performance of both reactors with chelated palladium is better than the homogeneous “control” variants in batch experiments and an open tube implementation both with 1 mol% pristine palladium complex Pd(MeCN)_2_Cl_2_ as a catalyst (with respect to the iodobenzene) in the same fluid phase (Fig. S4†). Yet, we calculated overall catalyst concentrations of 10 mmol L^–1^ (catalyst **1**) and 4 mmol L^–1^ (catalyst **2**) in the monolithic reactors (see the ESI†). This qualitatively indicates that at a reactant concentration of 0.1 mol L^–1^ in the fluid phase of one reactor volume, a 10 mol% of the palladium sites is present for catalyst **1**. Eventually, not all of the palladium sites in the material are catalytically active, which may originate from the limited accessibility of the very small pores providing diffusional resistance and significantly contributing to the large dry-state surface areas (Fig. S1†).

**Fig. 3:**
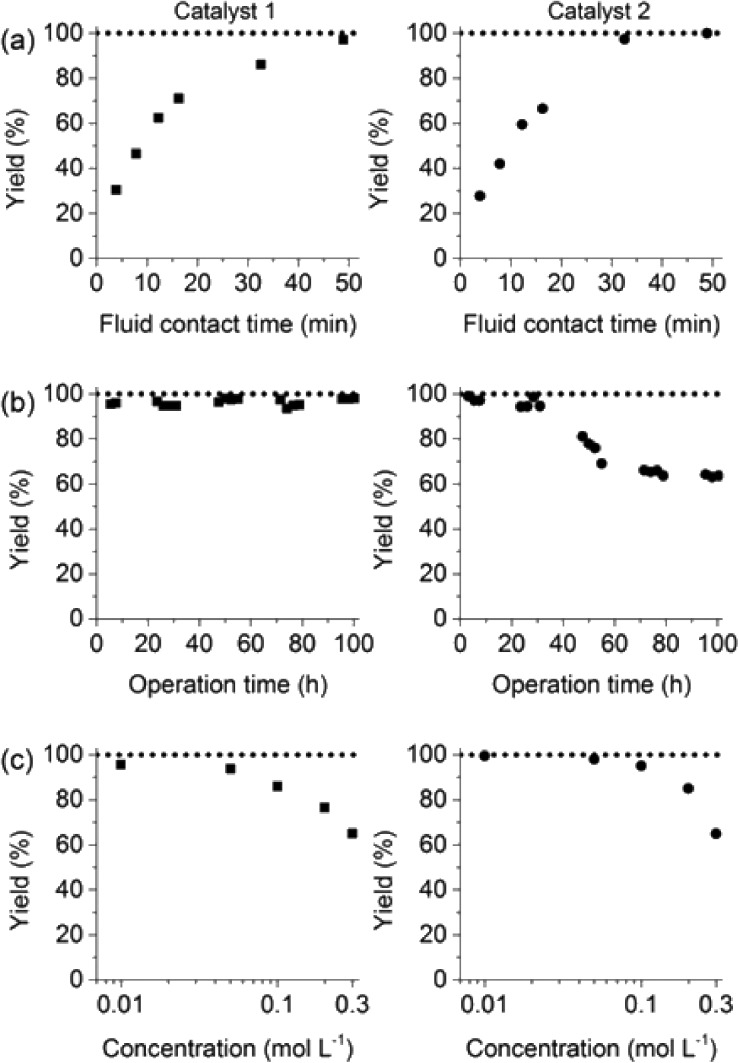
(a) Impact of fluid contact time in the reactors on yield obtained for the reaction of 0.1 mol L^–1^ iodobenzene with 0.125 mol L^–1^ *p*-tolylboronic acid (entry 1, Table 1). (b) Long term stability of the reactors in steady-state at a fluid contact time of 49 min. (c) Yield achieved from flow-through catalysis of the reaction of iodobenzene with *p*-tolylboronic acid keeping a stoichiometric ratio of 1 : 1.25 but varying concentration at a fluid contact time of 32.6 min. The reactors had the same length of 30 cm. Other conditions: fluid phase of 75/25 acetonitrile/water (%, v/v) and 2 equiv. triethylamine (to that of the iodobenzene) as the base. Reactions were performed at a constant reactor temperature of *T* = 80 °C.

Notwithstanding, the performance of the reactors could be deduced from a good distribution of flow and significant interactive contact area with chelated palladium functionality. We further found stability and robustness of the reactors over prolonged periods of time demonstrated for this example reaction using catalyst **1** for more than 122 reactor volumes and more than 100 hours of continuous operation (Fig. 3b). In contrast, catalyst **2** lost performance at longer times of operation, though initial steady-state reactor performance under these conditions is readily similar to both. These results strongly indicate the lower stability of catalyst **2** associated with the ligand linking strategy (Scheme 1). It is worth noting that the demonstrated fluid contact times in this experiment are smaller and the obtained yields larger than those reported in the literature.^8b,c^ Stability of the microscale reactor containing catalyst **1** as well is demonstrated for a timescale twice larger than that of a recently reported larger scale packed bed reactor.^6*d*^ Varying reactant concentrations over more than an order of magnitude for both reactor systems showed an expected, though decent, loss of quantitative yield at increased reactant concentration until the reactant solubility limit in the fluid phase is reached (Fig. 3c).

The catalytic system worked for other reactants as well and was studied for more stable catalyst **1**. The determined conversion and yields are summarized in Table 1. High selectivity for the catalyzed reaction with an exclusive absence of homocoupling of the aryl halide together with small concentrations of the boronic acid homocoupling product is observed (Table 1). The coupling reactions with that of electron-donating methoxy groups on the aryl halide improved yields, except for the methoxy group in *ortho* position. These observations may be explained by steric effects. We found that electron-withdrawing groups such as esters to decrease the respective yields. In comparison, the amine substituent in *para* position shows a good yield under otherwise similar reactions conditions. This brief study indicates a systematic insight into catalytic implementations involving varying reaction partners, made possible with the microscale reactors.

**Table 1:** Catalytic reactions performed in the 100 μm I.D. fused silica capillaries containing catalyst **1** operated with 75/25 acetonitrile/water (%, v/v). Concentration of each aryl halide was 0.1 mol L^–1^ and of each boronic acid 0.125 mol L^–1^ with 2 equiv. of triethylamine (to that of the aryl halide) as the base. The reactions were performed at *T* = 80 °C with a reactor fluid contact time of 32.6 min

Aryl halide	Boronic acid	Main product	%Yield^*a*^
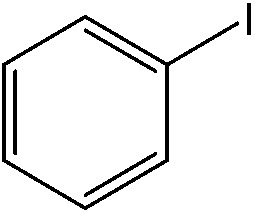	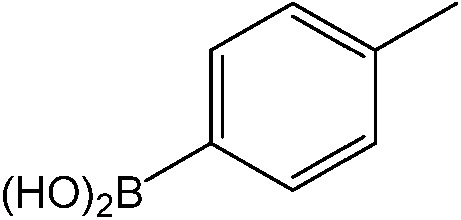	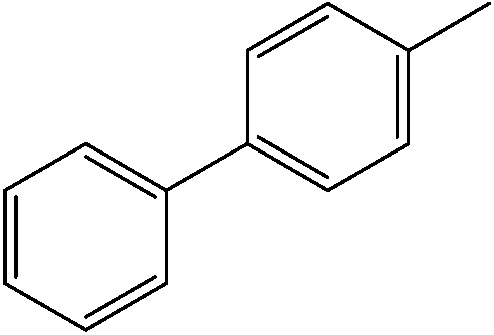	85
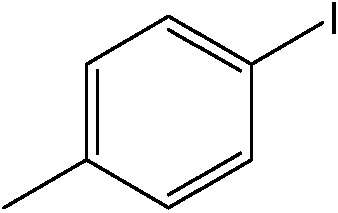	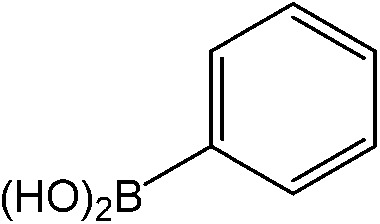	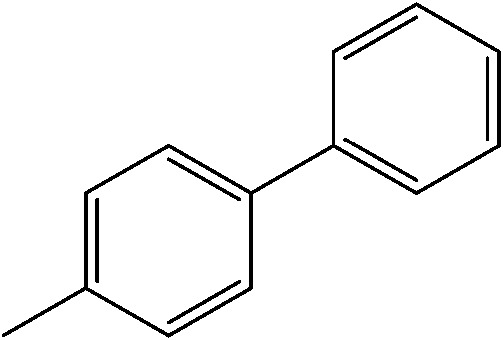	95
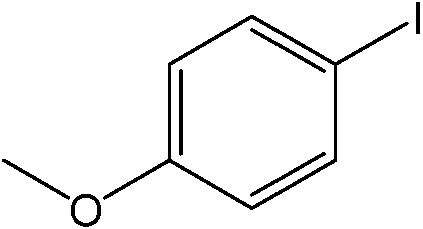	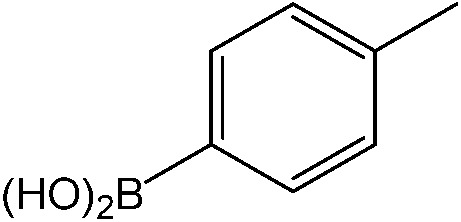	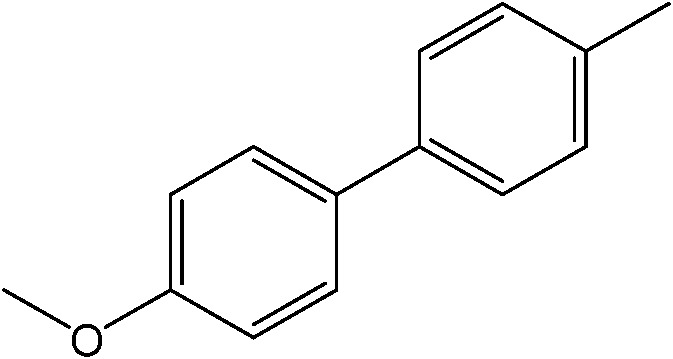	95
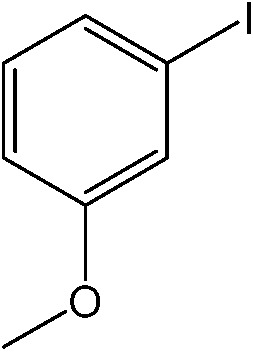	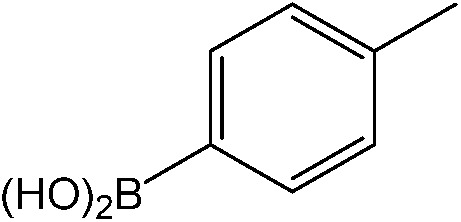	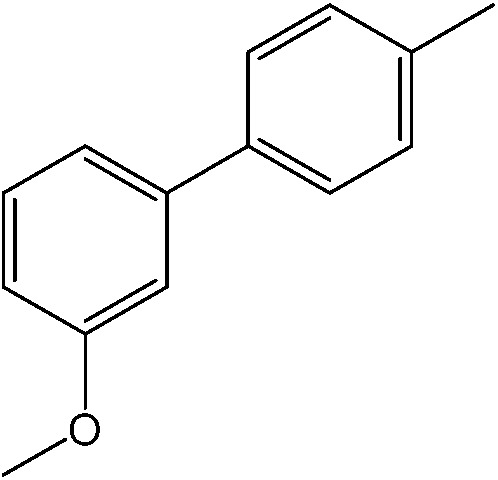	97
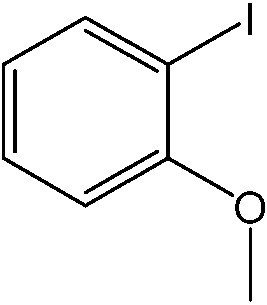	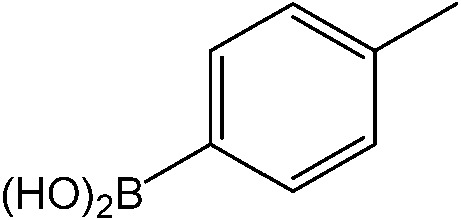	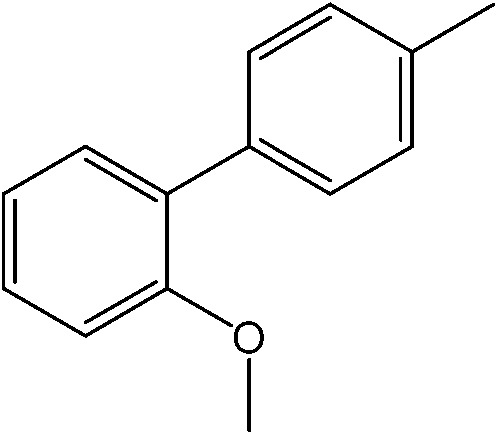	17
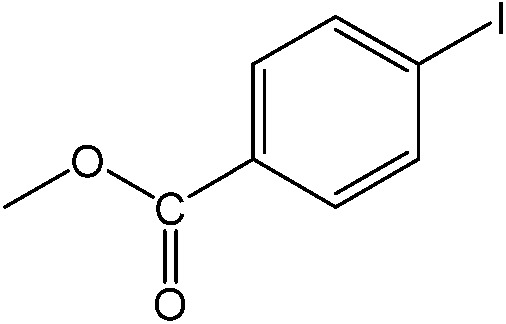	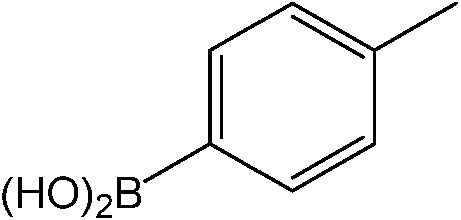	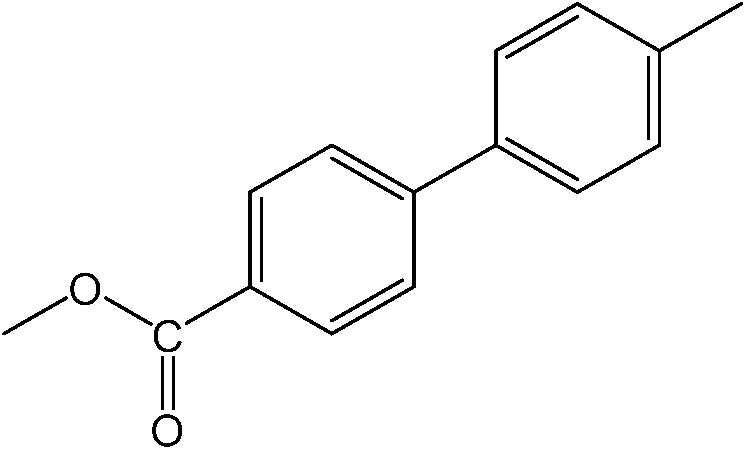	10
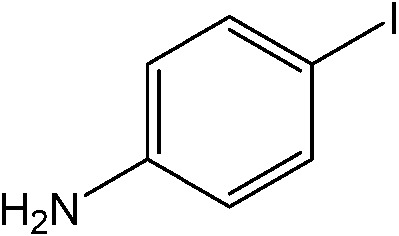	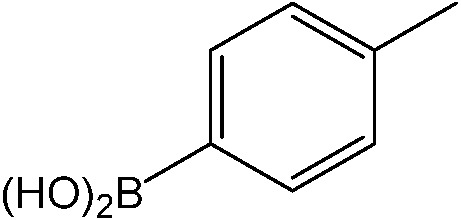	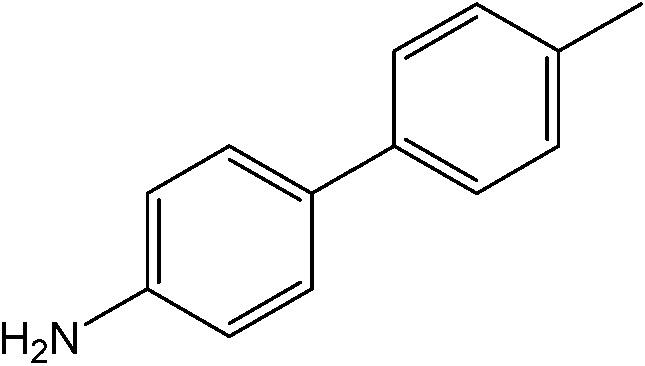	58

^*a*^Conversion of the aryl halide was found equivalent to the yield of main product; yield of homocoupling of the boronic acid was <1% in all cases.

In conclusion, the results presented in this preliminary account clearly demonstrate the possibility of the preparation of highly efficient and robust capillary flow reactors based on a large surface area, hierarchically-structured porous hybrid material constructed *in situ* from vinyl POSS. The excellent integration ability of this material and the *in situ* modification demonstrated here for the generation of a catalytically active chelated palladium functionality used for Suzuki and other carbon–carbon cross-coupling reactions is only one implementation these materials may find use in. Further tailoring of porous, hydrodynamic, and in particular chemical properties of the described pristine porous hybrid polymer in this article may enable further exploration of diversity and associated performance. The materials may pave the way for a family of catalyst systems which allow independent tailoring of porous and hydrodynamic properties as well as internal chemistry, both building a pivotal role for catalytic implementations.
