# Supplementary material for: Miniaturized catalysis: monolithic, highly porous, large surface area capillary flow reactors constructed in situ from polyhedral oligomeric silsesquioxanes (POSS)
Source: Catal Sci Technol. 2015 Jun 17;5(8):3917–21. doi: 10.1039/c5cy00510h (PMC4530614; doi:10.1039/c5cy00510h)
Supplement: Supplementary file 1 [file CY-005-C5CY00510H-s001.pdf]

Electronic Supplementary Material (ESI) for Catalysis Science & Technology.
This journal is © The Royal Society of Chemistry 2015

Supporting Information

Miniaturized Catalysis: Monolithic, Highly Porous, Large Surface Area Capillary Flow Reactors Constructed In situ from Polyhedral Oligomeric Silsesquioxanes (POSS)

*Pascal Scholder and Ivo Nischang**

Institute of Polymer Chemistry, Johannes Kepler University Linz, A-4060 Leonding, Austria, ivo.nischang@jku.at

Equipment

Scanning electron microscopy (SEM) and energy-dispersive X-ray spectroscopy (EDX) were performed with a Crossbeam 1540 XB electron microscope to probe the morphological dry-state properties of the prepared materials and existence of desirable chelated palladium functionality. Liquid chromatographic measurements were performed with a 1290 Infinity UHPLC instrument from Agilent Technologies (Agilent, Vienna, Austria). A packed, reversed-phase C18 silica-based chromatographic column (Rapid Resolution HD Eclipse Plus C18, 2.1 mm x 50 mm, particle size 1.8 µm) from Agilent (Vienna, Austria) was used for determination of conversions and yields.

Monolith modification procedures as well as catalytic reactions were all performed in flow-through mode by means of a syringe pump (Sigma-Aldrich, Vienna, Austria). Back pressure and porosity measurements of the pristine monolith were performed in 75/25 acetonitrile/water (%, v/v) with a nano-LC instrument (ThermoFischerScientific, Vienna, Austria) featuring a 4 nL injection valve and a UV detector with a 3 nL detection cell volume. For porosity measurements, uracil in 75/25 acetonitrile (%, v/v) was injected and the

porosity determined from its corrected elution volume and the calculated empty tube volume at a flow rate of 0.5 µL min^{-1}.[1]

Chemicals and Materials

The vinylPOSS® cage Mixture was purchased from Hybrid Plastics (Hattiesburg, USA) and azobisisobutyronitrile (AIBN) was received from ThermoFischerScientific (Geel, Belgium). Tetrahydrofuran, ethanol, acetone, methanol, and acetonitrile came from VWR (Vienna, Austria) while the hydrochloric acid, toluene, *N,N*-Dimethylformamide, and triethylamine came from J.T. Baker (Deventer, Netherlands). All other chemicals were purchased from Sigma-Aldrich (Vienna, Austria). All chemicals were used as received.

The 250 µL Hamilton glass syringes operated with the syringe pump were obtained from VWR (Vienna, Austria). Microfluidic connections were obtained from Idex Health & Science (Oak Harbor, USA). The polyimide-coated 100 µm I.D. fused silica-capillaries were from Optronis (Kehl, Germany).

Capillary wall functionalization

The empty capillary was first flushed with acetone and water. Then, it was flushed with 0.2 mol L^{-1} sodium hydroxide for one hour, followed by water, and subsequently 0.2 mol L^{-1} hydrochloric acid for 1 h, all with a flow rate of 2 µL min^{-1}. After flushing with water and ethanol, a 40 % 3-(Trimethoxysilyl)propyl methacrylate solution in ethanol (v/v) with an apparent pH value of 5 was pumped through the capillaries for two hours at a flow rate of 2 µL min^{-1}. Finally, the capillaries were flushed with ethanol, acetone, and dried under a stream of nitrogen.

Catalyst preparation

Catalyst 1. The vinylPOSS hybrid monolithic polymer was flushed with toluene. Then, a solution of 1.5 mL toluene, 88 µL 3-chloro-1-propanethiol, and 1.00 mg AIBN (1 wt% with respect to the thiol) was flushed through the monolith for 30 min at 1 µL min^{-1} and then with 0.16 µL min^{-1} for 20 h at a temperature of 80 °C to equip the internal surface with pendant chloro-functionality. Then, the reactor was flushed with toluene for 2 h with a flow rate of 1 µL min^{-1} followed by solvent exchange to methanol at a temperature of 60 °C. Subsequently, a filtered solution of 31.10 mg 5-amino-1,10-phenanthroline dissolved in 5 mL methanol was flushed through the monolith for 30 min at 1 µL min^{-1}, followed by 0.16 µL min^{-1} for 20 h at a temperature of 60 °C to allow for nucleophilic substitution (Scheme 1). Finally, the monolith was flushed with methanol to remove any unreacted 5-amino-1,10-phenanthroline.

Catalyst 2. The vinylPOSS polymer was flushed with a solution of 1 mg AIBN (1 wt% with respect to the thiol) and 75.5 µL thioglycolic acid in 1.0 mL tetrahydrofuran at 1 µL min^{-1} for 30 min, and 0.16 µL min^{-1} for a further 20 h, all at a temperature of 60°C. Afterwards, the capillary was washed with tetrahydrofuran at 1 µL min^{-1} for 1 h. To activate the pendant acid functionality, a solution containing 1.5 mL thionyl chloride, 1.5 mL tetrahydrofuran and 50 µL dimethylformamide was prepared. The monolith was flushed at a flow rate of 1 µL min^{-1} for 30 min at a temperature of T = 60°C, followed by flushing with tetrahydrofuran. After briefly flushing with methanol, a filtered solution of 31.10 mg 5-amino-1,10-phenanthroline in 5.0 mL methanol was pumped through the monolith at 1 µL min^{-1} for 30 min followed by 0.16 µL min^{-1} for 20 h at a temperature of 60°C. After amide bond formation, the reactor was flushed with methanol to remove any unreacted 5-amino-1,10-phenanthroline (Scheme 1).

Both phenanthroline-pendant scaffolds (Scheme 1) were flushed with acetonitrile and subsequently equipped with chelated palladium species. Therefore, a solution containing 15 mg $Pd(MeCN)_2Cl_2$ complex in 3.0 mL of acetonitrile was flushed through the monolith for at least 24 h at room temperature with a flow rate of 0.16 μL min^{-1}. Afterwards, the reactors were washed with acetonitrile to remove non-chelated palladium.

UV absorption measurements of solutions of $Pd(MeCN)_2Cl_2$ in acetonitrile before and after being pumped through the reactor (250 μL) were used to qualitatively estimate the amount of palladium retained by the material.

Catalytic reactions

Catalytic reactions were all performed in 75/25 acetonitrile/water (%, v/v). Triethylamine with 2 eq. to that of the aryl halide was used as a base. Catalytic reactions in the monoliths were all performed by means of a syringe pump allowing for modification of flow rates over desirable ranges from nano- to microflow. The reaction mixtures, containing toluene or benzene as an internal standard, were continuously delivered to the reactor through a 100 μm I.D. capillary while the outlet of the reactor was connected to a 100 μm I.D. capillary placed through a septum in the 1.5 mL glass vials, where the effluent was collected (Figure S3a).

For comparison, we utilized an empty equivalently-dimensioned fused silica-capillary tube of 100 μm ID (Figure S3b). The reactor inlet was equipped with a T-piece. Two syringes operated at the exactly same flow rates were utilized for catalytic reactions. One syringe contained the model reactant mixture in 75/25 acetonitrile/water (%, v/v) with triethylamine (2 eq. to that of the aryl halide) while the other contained $Pd(MeCN)_2Cl_2$ catalyst in 75/25 acetonitrile/water (%, v/v). In both instances (monolithic *vs* open tube) contact of reactants with the catalyst effectively took place at the inlet of the reactor (Figure S3 a, b).

Homogeneous batch solution experiments were performed in 75/25 acetonitrile (%, v/v) with equivalent reactant solutions as in flow with Catalysts 1 and 2. We utilized 0.01, 0.1, and 1 mol% $Pd(MeCN)_2Cl_2$ as the catalyst in closed 4 mL glass vials. The reaction was performed at a temperature of 80 °C and a stirring rate of 240 rpm.

The effluent/samples from all reactions performed were appropriately diluted, and analyzed offline by means of liquid chromatography. Aqueous acetonitrile was used as the mobile phase. We utilized a linear gradient elution at a flow rate of 0.5 mL min^{-1} and a gradient program starting from 30 % acetonitrile in water (v/v) to 80 % acetonitrile in water (v/v) within 3 minutes, a constant hold for 1 minute, before ramping back to injection conditions within 3 min.

References

1 I. Nischang, F. Svec, and J. M J. Fréchet, *J. Chromatogr. A*, 2009, **1216**, 2355–2361.

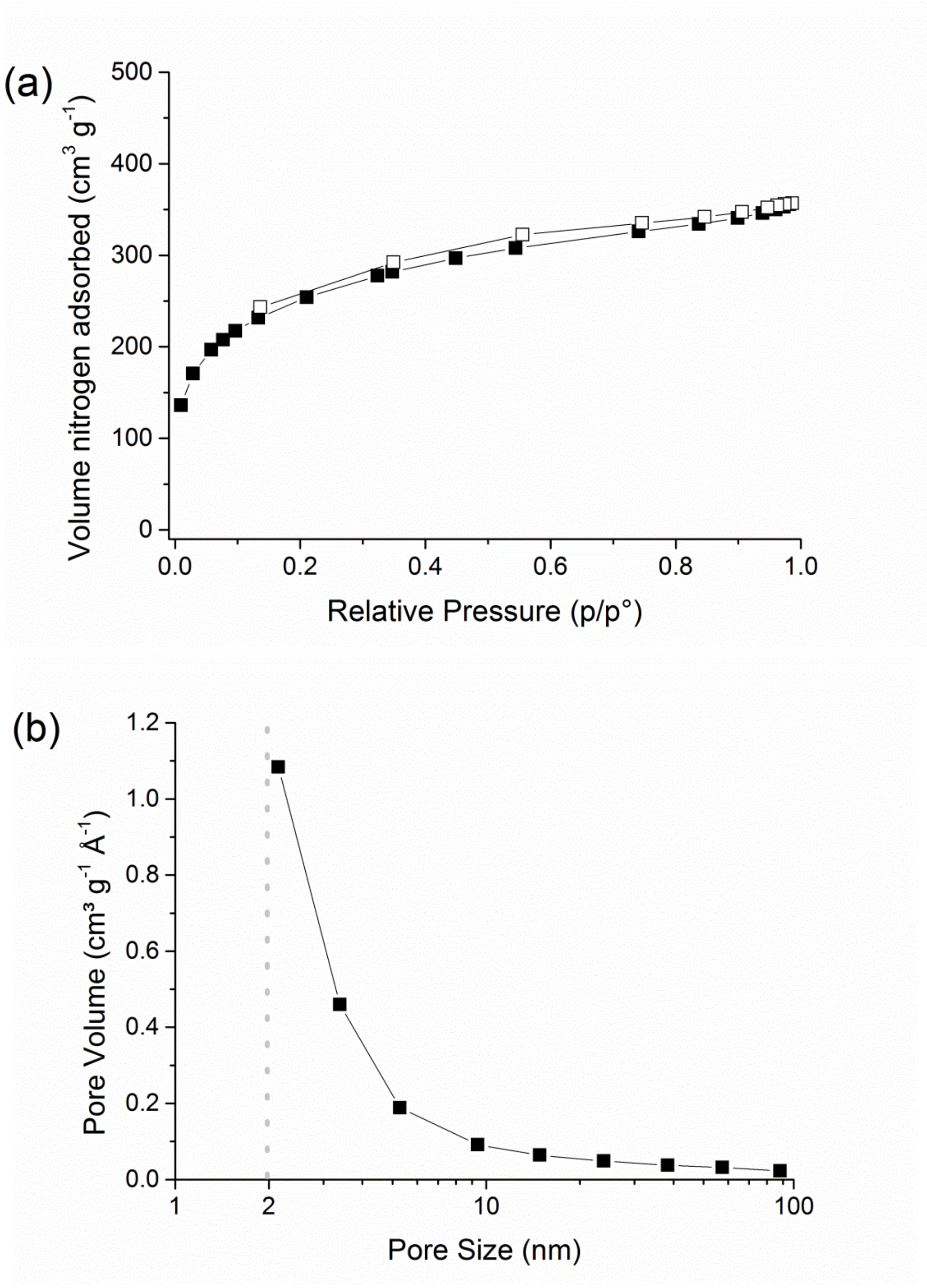

Figure S1. (a) Nitrogen adsorption/desorption isotherm of bulk material prepared in 4 mL glass vials under otherwise same conditions as for the microflow reactors and (b) Barrett-Joyner-Halenda (BJH) pore size distribution derived from the adsorption branch of the isotherms. The BET surface area of the materials was estimated as 898 $m^2\ g^{-1}$ with BJH mesopore volumes of 0.4 $cm^3\ g^{-1}$.

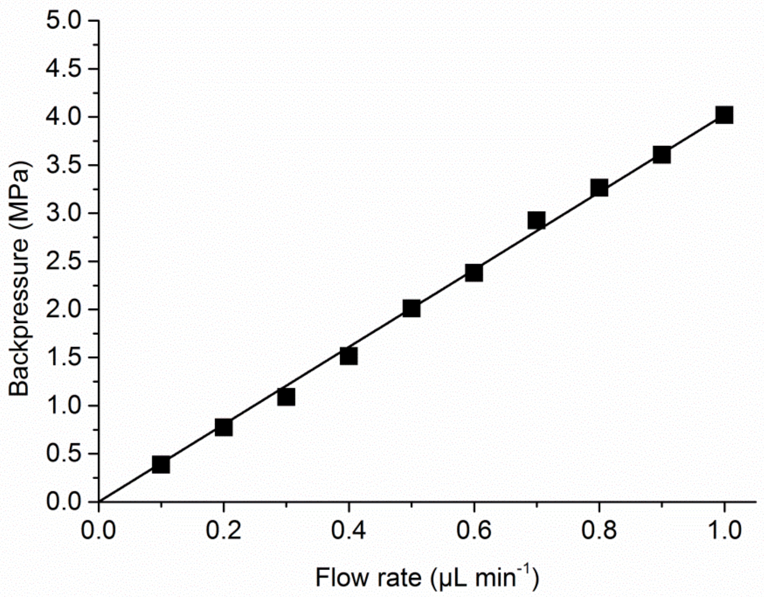

Figure S2. Plot of backpressure against flow rate recorded by the nano-LC instrument for an installed 32.5 cm long, 100 μm ID fused silica capillary containing the hybrid monolith pristine material. The mobile phase was composed of 75/25 acetonitrile/water (%, v/v). The solid line represents a linear fit to the data indicating that the materials show mechanical stability at increased flow rates. The largest flow rate used in the current study generates a decent backpressure of less than 5 Mpa, indicative of the materials low flow resistance.

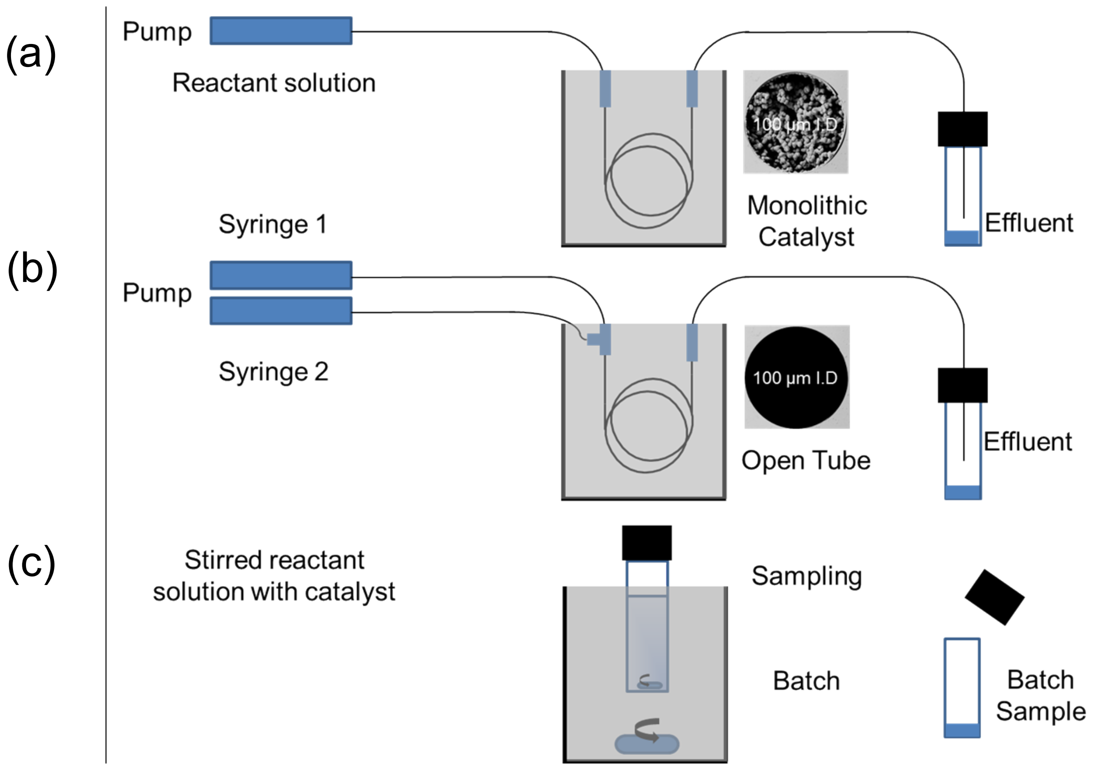

Figure S3. Experimental setup for catalytic experiments. (a) For heterogeneous catalysis the reaction mixture in 75/25 acetonitrile/water (%, v/v) containing triethylamine as the base was continuously pumped through the reactor containing chelated palladium (Scheme 1). (b) For homogeneous flow catalysis the reaction mixture in 75/25 acetonitrile/water (%, v/v) containing triethylamine (2 eq. to that of the aryl halide) as the base (Syringe 1) and the catalyst at 2 mol% in 75/25 acetonitrile/water (Syringe 2) were both continuously delivered to the open tube reactor through a mixing T-piece. For both implementations (a) and (b) the reactors had an equivalent length of 30 cm. (c) Homogeneous batch implementation of the catalytic reaction in 75/25 acetonitrile/water containing triethylamine (2 eq. to that of the aryl halide) as the base and a catalyst concentrations of 0.01, 0.1, and 1 mol%.

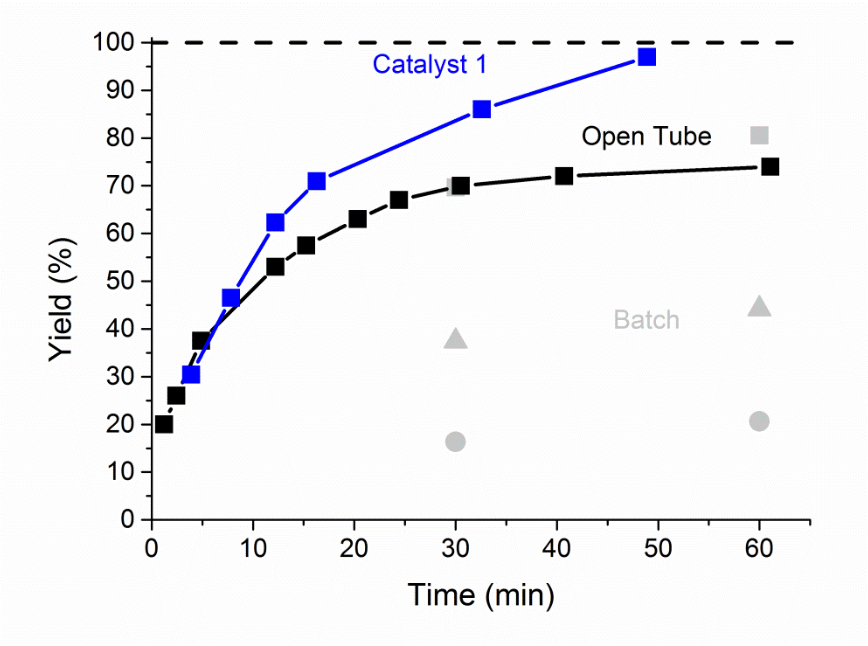

Figure S4. Comparison of quantitative yield of homogeneous batch experiments at three different catalyst concentrations of 0.01 (gray circles), 0.1 (gray triangles), and 1 mol% (gray squares), the homogeneous open tube implementation (black squares), and **Catalyst 1** (blue squares). Concentration of iodobenzene was 0.1 mol L^{-1} and of *p*-tolylboronic acid 0.125 mol L^{-1} with 2 eq. of triethylamine (to that of the iodobenzene) as the base. Please note that for flow implementations (Figure S3 a, b) contact with the catalyst took place at the inlet of the reactor.
